# Supplementary material for: Inventive nesting behaviour in the keyhole wasp Pachodynerus nasidens Latreille (Hymenoptera: Vespidae) in Australia, and the risk to aviation safety
Source: PLoS One. 2020 Nov 30;15(11):e0242063. doi: 10.1371/journal.pone.0242063 (PMC7703898; doi:10.1371/journal.pone.0242063)
Supplement: Attachment — Submitted filename: Responses to Reviewers.docx [file pone.0242063.s001.docx]

Responses to Reviewers

Reviewer #1: Dear Authors, I found your study very interesting and Ia also realize the urgency to publish this MS as it can help preventing accidents in airports.

I have made several comments and suggestions to enhance the quality of the MS, and I am especially concerned with the data and analyses performed. You have such rich data but they were not explored in its full magnitude. I also reommended several changes in the Methods section, to make the MS replicable.

Specific comments:
L 21 - 22 please rephase this sentence

Sentence has been rephrased and punctuation added.

Introducion - you need to work hard on your objectives and hypotheses (you can have hypotheses). As I read you results, I realized that you introduction (is good) lacks more robust objectives. You have investigated so many things, such as sex rations, influence of weather, environment and etc, and these factors are not presented in the Introduction.

We have added some simple questions, rather than hypotheses, to indicate the aims more clearly and justify the analyses. We feel that as so little is known on this topic that the question approach is more valid.

L 57 - a parenthesis before Florida, but it does not close after Arizona (?)

Closing parenthesis added.

L 163 - sample size = 12, correct? Or is there anymore treatments? How many probes per sheet? From your figures, I counted 4 probes in one sheet and five probes in other sheet. Did you use all probe types (Table 1) in the experiment?

All probe types were used, but not all probe types in all locations. For analyses that included location, only probes deployed at all locations were used.

L 164 - incidents .... were suspected

Text has been amended.

Intercept traps - what is the sample size? For instance, you used 16 traps in airside and each trap consisted of 68 tubes, so in this example you sample size is 68x16 = 1088 tubes? Is there another source of variation? Please provide these number in the MS, as it helps to understand your statistical analyses. Moreover, you mention that tubes were replaced weekly, but it is not clear for how long.

This paragraph has been amended to make the design clearer.

Monitoring - here again I miss quantitative information. "Probes were inspected weekly to monthly" - all probes? how many times were they inspected during the study period?

Yes, all probes were examined each time. They were checked 49 times between the Feb 16 and April 19 dates. Text has been amended to make this clearer.

"Blocked probes were removed from the panels" - were these probes replaced by now ones?

Yes. Text has been added.

Data Analysis - I think you should explore the data with the following tests. Please check them to see if they are appropriate to the data you collected. I think using chi-squared tests are a poor indicator of statistical power.

We have applied more rigorous tests to the data where appropriate, for instance a generalised linear mixed model (glmm in R) to explore relationships between blockages (fixed effect) and location and probe type (random effects). We improved the accuracy of this analysis by only including probe types that were deployed at all locations on the airport. However, we have retained the overall approach of separate analyses (rather than the complex model fitting suggested below), as these allow us to answer each of the questions posed in the Introduction directly. Note that we have made the sex ratio test more robust by using the Cochran-Armitage trend test rather than chi square – with the same result.

lmm, glmm, or glmmTMB = number of occupied probes ~ location (4 locations) + probe size/volume/apperture + probe height + weather + time + (1|panels)

lm or Anova = occupied traps ~ location (2 locations) + time

See above. GLMM used for blockages ~ location + probe type, but not time as this is explored elsewhere.

L 196 - 201 - this shoud be on Methods. We are unclear what the reviewer is requesting here – this section is already contained within the Methods section.

L 313. a dot is missing after P (wasp genus).

Period added.

L 202 - I did not understand which variables you used in this multiple regression. What as the intercept, the number of wasps?

The regressions are between the number of blockages (i.e. nests) and proportions of the different habitat types at increasing distances from the panels. The text has been amended to make this clearer.

Results
L 208 - only here I see the duration of the study. I did not understand this number of 93 blocked probes. In the Methods section,
you need to provide more information on sample sizes. 93 probes correspond to what percentage of probes available? Here I see as well that there is a time variable, and also a weather variable. From what you presented here, I cannot reverse the results to replicate a methodology.

Have added text to Methods to indicate duration of the study.

Figure 3 - I suggest presenting the data in percent values, because you mention in the MS that you did not have a perfectly balanced design.

We believe raw numbers are correct, as there were always the same number of probes available for nesting. Converting to percentages would be redundant.

L 231 - 233 - There must be problems with this Anova. I suggest changing the test (as I recommended above).

We have incorporated this analysis into the mixed model of location and probe type (see above), and re-written the interpretation accordingly.

Figure 4 - these relations are not linear, and in Fig 4a there is an outlier in the data. Moreover, the text displays a value o R = 0.73, e the figure it is 0.53.

Text has been amended. In the text R was quoted, and on the figures R^2^. The relationship in Fig.4a is significant despite the outlier.

Reviewer #2: This is an interesting study on the nesting ecology of a solitary wasp, Pachodynerus nasidens (Eumeninae). The authors made an innovative experiment based on a real problem frequently observed in airports. The study is important since provides the natural history aspects of the wasp and explores the preference of nesting sites which are the aircraft model pitots. The results are well explored but they used parametric ANOVA, so it is to state whether samples have normal distribution or may they transformed the raw data. Also, the data are not available.

Data has been saved to OSF doi: 10.17605/OSF.IO/T87ZY.

We have no reason to expect the data to be anything other than normally distributed, so parametric analyses are valid. We could reasonably expect that any probe could be blocked at any location on the airport at any time.

The authors may suggest a further comment for companies to modify size or shape(?) of pitots since they showed a preference of probe aperture diameter between 4-7.2 mm.

We have added a comment about this and a reference to our other paper (now published) which discusses this in more detail.
