# Supplementary material for: Inventive nesting behaviour in the keyhole wasp Pachodynerus nasidens Latreille (Hymenoptera: Vespidae) in Australia, and the risk to aviation safety
Source: PLoS One. 2020 Nov 30;15(11):e0242063. doi: 10.1371/journal.pone.0242063 (PMC7703898; doi:10.1371/journal.pone.0242063)
Supplement: Attachment — Submitted filename: Responses to reviewers.docx [file pone.0242063.s002.docx]

Responses to Reviewers


Reviewer #1: Dear authors, I appreciate a lot the polite responses regarding the first row of peer review. I still have some minor doubts/suggestions:

Please cite the R packages used in the study

*Have updated citations to both R packages used.*

How did you collect the data of climatic variables?

*Detail and reference added.*

Why including location as a random variable? I believe this is a fixed factor

*We have retained location as a random variable as we had no a priori assumption or knowledge about where on the airport wasps were nesting.*

L 234 - I did not understand the "Monte Carlo sample size". Is this misplaced?

*Have changed sample size to number of simulations and added text – these are run as part of glmm to derive the most stable maximum likelihood results.*

L 244 - 249 and Table 2 - these are Methods (above) and not Data Analyses

*Text and table moved to Methods*.

L 261 - 264 - please prove the R² values

*Have changed these R values to adjusted R and added ANOVA results for proof.*

L 281 - The mixed effects model confirmed probe type was not significant but then you mention that they were significant.
This is a bit confusing. I suggest stating that probes were significant, because above you also show that probes > 5 mm were found to have nests.

*We agree this is bit confusing. The differences arise because for the formal test we could only use probe types that were deployed at all locations – this left out the smaller probes, so the result was not significant*

L 351 - 354 - are these r² values based on which test? Did you perform a regression here or is this part of the sex ratio test (Cochran-Armitage test).

*Yes this is a result from the Cochran-Armitage test for trends. Have added text to indicate this.*

IF you are having difficulties to express the data collection and analyses, I suggest making a table like Table 1 (with you adaptations) made in the following papers. It helps a lot to understand the tests and figures associated with each objective.

*Have added Table 3. Thanks you for this suggestion!*

Figure 4a - there is no need for this Figure

*This figure has been removed*

*We have also made minor improvements to the text, also as tracked changes.*
